# Dissociation, cognitive reflection and health literacy have modest effect on belief in conspiracy theories about COVID-19

**DOI:** 10.1192/j.eurpsy.2022.531

**Published:** 2022-09-01

**Authors:** V. Pisl, J. Vevera

**Affiliations:** 1 Faculty of Medicine and University Hospital in Pilsen, Charles University, Department Of Psychiatry, Plzen, Czech Republic; 2 Faculty of Medicine in Pilsen, Charles University, Department Of Psychiatry, Plzen, Czech Republic

**Keywords:** conspiracy theories, conspiracy mentality, Covid-19, health literacy

## Abstract

**Introduction:**

Understanding the predictors of belief in covid-related conspiracy theories and willingness to get vaccinated against COVID-19 may aid the resolution of current and future pandemics.

**Objectives:**

We aim to investigate how psychological and cognitive characteristics influence general conspiracy mentality and covid-related conspiracy theories.

**Methods:**

A cross-sectional study was conducted based on data from an online survey of a sample of Czech university students (n=866) collected in January 2021, using multivariate linear regression and mediation analysis.

**Results:**

Sixteen percent of respondents believed that COVID-19 is a hoax; 17% believed that COVID-19 was intentionally created by humans. Seven percent of the variance of the hoax theory and 10% of the variance of the creation theory was explained by (in descending order of relevance) low cognitive reflection, low digital health literacy, high experience with dissociation and, to some extent, high bullshit receptivity. Belief in covid-related conspiracy theories depended less on psychological and cognitive variables compared to conspiracy mentality (16% of the variance explained). The effect of digital health literacy on belief in covid-related theories was moderated by cognitive reflection.

**Conclusions:**

Belief in conspiracy theories related to COVID-19 was influenced by experience with dissociation, cognitive reflection, digital health literacy and bullshit receptivity.

**Disclosure:**

No significant relationships.

